# Proteasome Dysfunction Leads to Suppression of the Hypoxic Response Pathway in Arabidopsis

**DOI:** 10.3390/ijms232416148

**Published:** 2022-12-18

**Authors:** Xue Xia, Chun-Meng Tang, Gu-Zi Chen, Jia-Jia Han

**Affiliations:** 1 Yunnan Key Laboratory of Plant Reproductive Adaptation and Evolutionary Ecology, Institute of Biodiversity, School of Ecology and Environmental Science, Yunnan University, Kunming 650500, China; 2 State Key Laboratory for Conservation and Utilization of Bio-Resources in Yunnan, Yunnan University, Kunming 650500, China

**Keywords:** Arabidopsis PBE1, proteasome dysfunction, hypoxia response, waterlogging stress, root meristem, shoot meristem, re-plication

## Abstract

Proteasome is a large proteolytic complex that consists of a 20S core particle (20SP) and 19S regulatory particle (19SP) in eukaryotes. The proteasome degrades most cellular proteins, thereby controlling many key processes, including gene expression and protein quality control. Proteasome dysfunction in plants leads to abnormal development and reduced adaptability to environmental stresses. Previous studies have shown that proteasome dysfunction upregulates the gene expression of proteasome subunits, which is known as the proteasome bounce-back response. However, the proteasome bounce-back response cannot explain the damaging effect of proteasome dysfunction on plant growth and stress adaptation. To address this question, we focused on downregulated genes caused by proteasome dysfunction. We first confirmed that the 20SP subunit PBE is an essential proteasome subunit in Arabidopsis and that PBE1 mutation impaired the function of the proteasome. Transcriptome analyses showed that hypoxia-responsive genes were greatly enriched in the downregulated genes in pbe1 mutants. Furthermore, we found that the pbe1 mutant is hypersensitive to waterlogging stress, a typical hypoxic condition, and hypoxia-related developments are impaired in the pbe1 mutant. Meanwhile, the 19SP subunit rpn1a mutant seedlings are also hypersensitive to waterlogging stress. In summary, our results suggested that proteasome dysfunction downregulated the hypoxia-responsive pathway and impaired plant growth and adaptability to hypoxia stress.

## 1. Introduction

The proteasome also named the 26S proteasome, is a large proteolytic complex that consists of two subcomplexes: the catalytic 20S core particle (20SP) and the 19S regulatory particle (19SP) in eukaryotes [1,2]. Generally, the proteasome degrades the target protein modified by ubiquitination through the ubiquitin-proteasome pathway [1,3]. Meanwhile, the proteasome also directly degrades protein substrates in a manner independent of ubiquitin [4] or 19SP [5]. In eukaryotes, proteasomes degrade most cellular proteins, thereby controlling many key processes, including gene transcription, RNA metabolism, and protein quality control [3,4,6,7,8,9]. Proteasome-mediated degradation is vital in eukaryotic cells and organisms, and dysfunction of the proteasome is associated with diverse human diseases, such as cancer and neurodegeneration [3,10]. Meanwhile, different subunits of the proteasome have been shown to be important for plant development and stress responses, such as RPN1a [11,12,13,14], RPN5a [15], RPN10 [16,17], RPT2a [18,19,20], RPT12a [21], PBE1 [22] and PAG1 [4,23]. However, most of these studies only focused on different subunits of 19SP. Considering that 20SP can directly degrade some important proteins containing unstructured regions, a stable mutant of an essential 20SP subunit would be useful to explore the effects of proteasome dysfunction on plant development and stress responses.

20SP, which performs essential functions in both ubiquitin-dependent and ubiquitin-independent pathways, is assembled in a pseudo-sevenfold symmetry (α_1-7_β_1-7_β_1-7_α_1-7_). Among them, three β subunits are proteolytically active: PBA (β_1_), PBB (β_2_), and PBE (β_5_) [1]. Loss-of-function mutation in PBE, but not PBA or PBB, is lethal in yeast [2,24], demonstrating that the PBE subunit is essential for proteasome functions. In humans, in addition to the standard PBE subunit β_5_, there are two tissue-specific forms/types of PBE subunits: β_5_i of the immunoproteasome and β_5_t of the thymus-specific proteasome [3]. In Arabidopsis and tomato, environmental stress conditions induce changes in the composition or modification of the β5 subunit [22,25]. In the Arabidopsis plant, there are two paralogous β5 subunits (PBE1 and PBE2), and PBE1 plays an important role in proteasome assembly and the plant response to salt stress [22]. By modulating the protein level of the transcription factor abscisic acid insensitive 5 (ABI5) in the ubiquitin-dependent proteasome degradation pathway, PBE1 indirectly regulated the expression of downstream responsive genes under salt stress [22]. Meanwhile, 20SP can degrade SERRATE (SE), a key factor of RNA metabolism containing unstructured regions, through a ubiquitin-independent proteasome degradation pathway in Arabidopsis [4]. *PBE1* mutation leads to the accumulation of SE protein, suggesting the role of PBE1 in RNA metabolism and gene expression [4]. In this study, we used a stable loss-of-function mutant of Arabidopsis *PBE1* to explore the effects of proteasome dysfunction on gene expression profiles and their function in plant development and stress response.

## 2. Results

### 2.1. Mutation in PBE1 Causes Proteasome Dysfunction in Arabidopsis Seedlings

The β5 subunit of 20SP has two paralogs (PBE1 and PBE2) in Arabidopsis. We analyzed the genotype of self-crossed progeny from the *pbe1 pbe2* heterozygous double mutant (AaBb) and failed to identify plants that were homozygous for both (aabb) or plants homozygous for one mutation and heterozygous for the other (Aabb or aaBb) (Table 1 and Table S1). The absence of these allelic combinations is consistent with the inviability of *pbe1 pbe2* double mutant gametes. To further confirm this result, we conducted reciprocal crosses using pollen from WT plants to fertilize the ovules of *the pbe1 pbe2* double heterozygous mutant and vice versa. The genotyping of the resulting progeny in each cross failed to identify *pbe1 pbe2* double heterozygous (AaBb) individuals (Table 2). These data further confirmed that the simultaneous loss of *PBE1* and *PBE2* is lethal, and the proteasomal β5 subunit is essential for the survival of both gametophytes and sporophytes in Arabidopsis.

**Figure 1:**
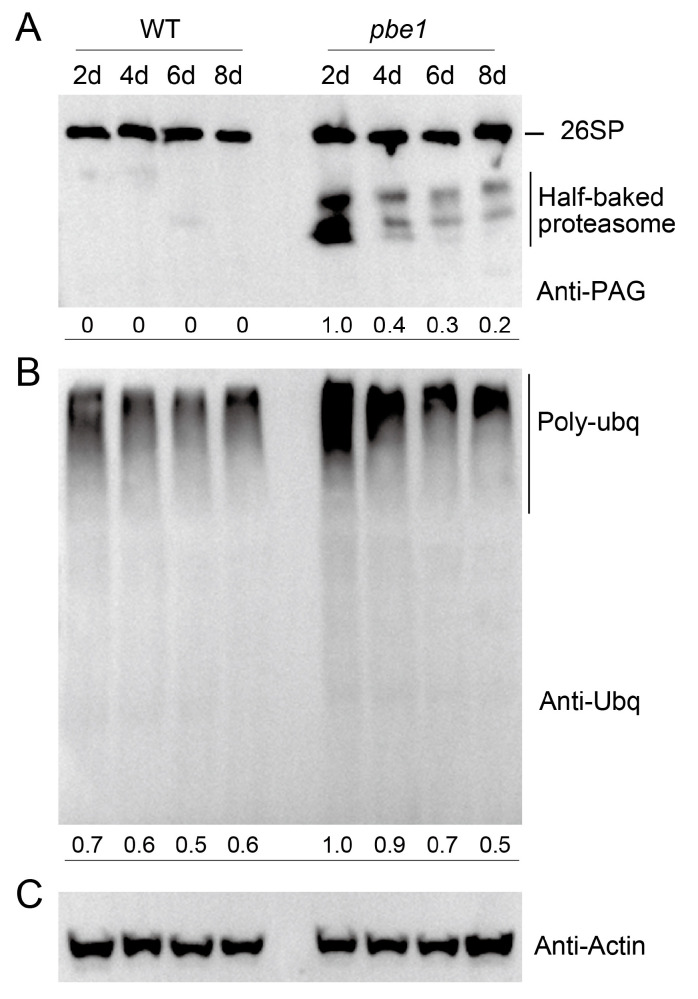
Mutations in *PBE1* cause Arabidopsis proteasome dysfunction. Proteasome assembly and protein polyubiquitination levels were detected in the wild-type (WT) and *pbe1* mutant plants, which were grown on ½ MS media for 2, 4, 6, or 8 days. (**A**) Proteasome assembly was detected via native PAGE with an anti-PAG1 antibody. (**B**) Polyubiquitination of total protein was detected by SDS-PAGE and Western blotting with an anti-ubiquitin (Ubq) antibody. The numbers below indicate the relative abundance relative to the loading control (actin). (**C**) Actin was used as the loading control.

In a previous study, we demonstrated that the expression of PBE1 is much higher than that of PBE2 in wild-type (WT) plants under normal growth conditions, and the total β5 subunit was greatly reduced in the *pbe1* mutant (*pbe1-2*) but not in the *pbe2* mutant (*pbe2-1*) [22]. To investigate the effect of PBE1 mutation on the proteasome, we first detected proteasome assembly in *pbe1* mutant plants grown under normal conditions (Figure 1). Proteasome assembly experiments of 2-, 4-, 6-, and 8-day-old seedlings revealed that ‘half-baked’ proteasomes were produced in large numbers in the *pbe1* mutant, especially in younger seedlings, and no ‘half-baked’ proteasome was produced in WT (Figure 1). Polyubiquitinated proteins are degraded by the proteasome degradation pathway. As expected, the polyubiquitinated proteins of the *pbe1* mutant were also markedly higher than those of WT seedlings (Figure 1). In short, the proteasomal function is impaired in the *pbe1* mutant.

Considering that the β5 subunit is required for both ubiquitin-dependent and ubiquitin-independent proteasome degradation pathways, the *pbe1* mutant (pbe1-2, SALK_092686) is used in subsequent experiments to study the effects of proteasome dysfunction on plant development.

### 2.2. RNA-Seq Analysis of Proteasome Dysfunction-Induced Regulation

**Figure 2:**
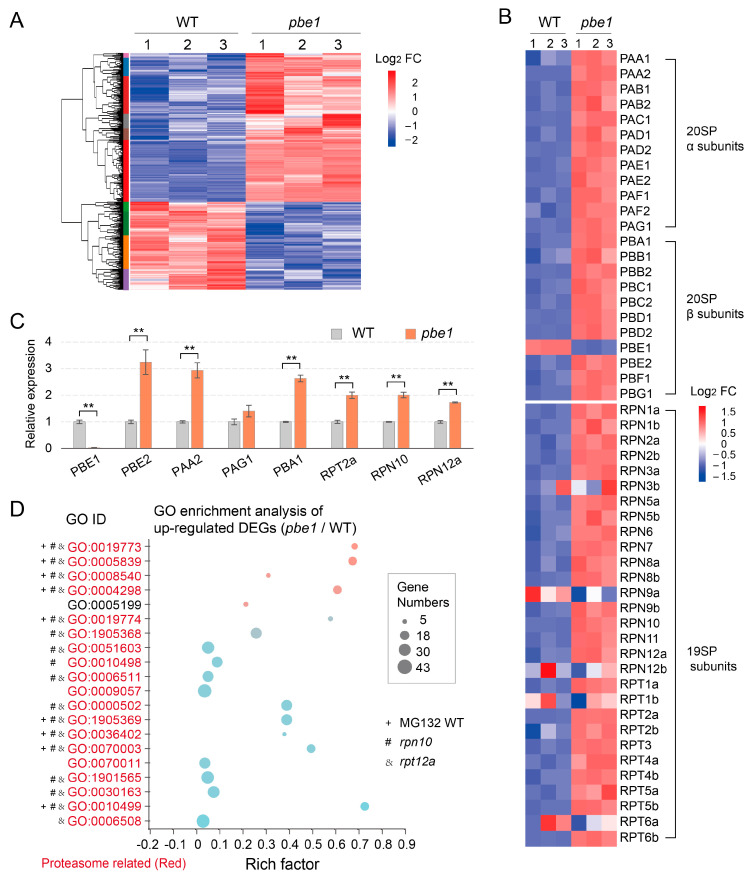
RNA-Seq analysis of the *pbe1* mutant. (**A**) Heat map showing the expression patterns of the up- and down-regulated differentially expressed genes (DEGs) (absolute log2 fold-change ≥ 1 and Q-value ≤ 0.05) in the *pbe1* mutant compared to WT plants. Three biological replicates were conducted for each sample. (**B**) Heat map showing gene expression patterns of the proteasomal subunits in the *pbe1* mutant compared to WT. Red and blue represent the relative expression of genes among different samples in the same row. (**C**) The expression of several key proteasome subunit genes was detected by RT-qPCR. The expression level of each gene was firstly normalized to that of the housekeeping gene *PP2A* or *ACTIN*. Then, the expression level in WT was set to one, and the expression level in *pbe1* was compared with that in WT to obtain the relative expression level. Data are the mean ± SD, n = 4. Significance analysis of differences was performed by *t*-test (** p < 0.01). (**D**) Gene Ontology (GO) analysis of upregulated DEGs showed that GO terms associated with proteasome were enriched. The marked GO terms were also enriched in upregulated DEGs in *rpn10*, *rpt12a* mutants, or MG132-treated WT seedlings (see Table S2 for details of the enriched GO terms of upregulated DEGs).

In plants and other eukaryotes, a large number of developmental and stress response signal proteins are degraded through proteasome degradation pathways [3,6,26]. Therefore, proteasome dysfunction must indirectly alter some developmental signaling pathways. Here, we compared the transcriptional profiles of WT and *pbe1* mutant plants under normal growth conditions and found 244 upregulated (*pbe1* divided by WT) DEGs and 144 downregulated DEGs (absolute log2 fold-change ≥ 1 and Q-value ≤ 0.05) (Figure 2). GO analysis of upregulated DEGs showed that the enriched GO terms were related to the proteasome or its related protein degradation pathways, except for one GO term, “cell wall structural component (GO:0005199)” (Figure 2 and Table S2). The expression heat maps and RT-qPCR analysis both confirmed that the expression of most proteasome subunit genes was upregulated in the *pbe1* mutant compared to the WT (Figure 2). In fact, the upregulation of proteasome subunit genes was also observed in 19SP subunit mutants (*rpn10*, *rpt12a*) or MG132 (a proteasome inhibitor)-treated wild-type seedlings [27]. Meanwhile, we reanalyzed the transcriptome data of *rpn10-* and *rpt12a*-mutant and MG132-treated WT plants in [27] and found that many enriched GO terms were similar to those in the *pbe1* mutant (Figure 2 and Table S2). In summary, by using RNA-Seq analysis of the *pbe1* mutant, we further demonstrated that plant cells would feedback activate the expression of proteasome subunit genes during proteasome dysfunction.

### 2.3. Proteasome Dysfunction Leads to Suppression of Hypoxia-Responsive Genes

**Figure 3:**
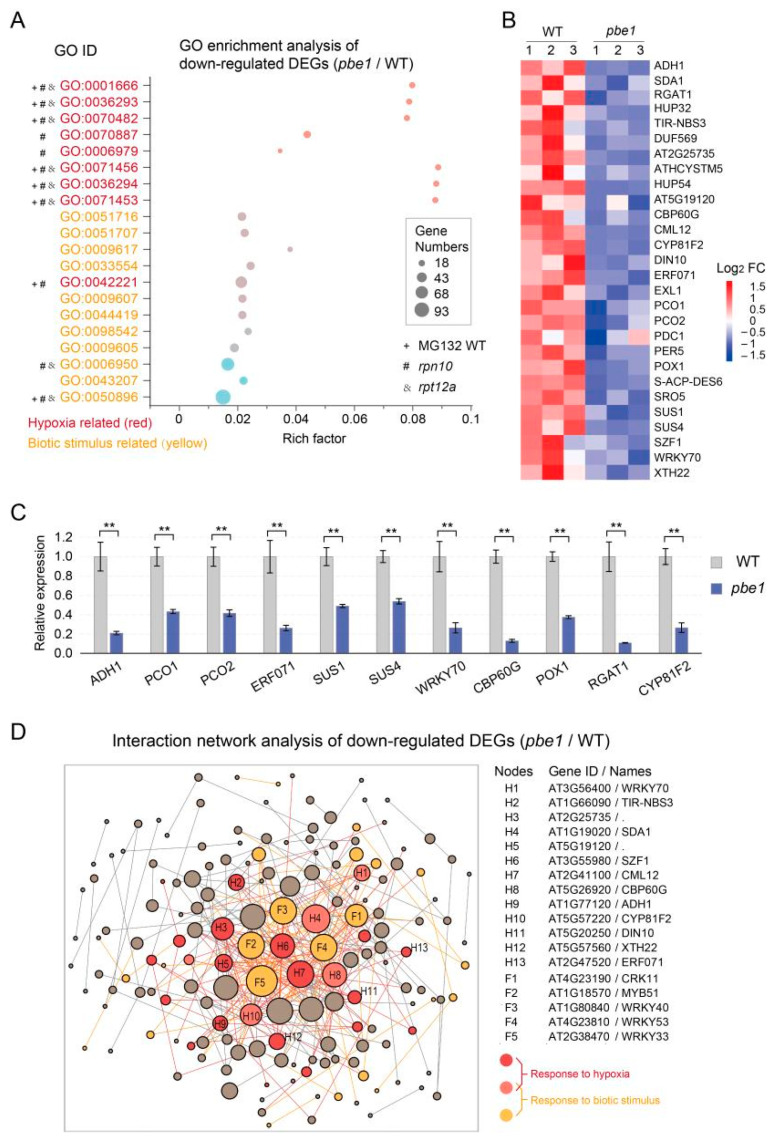
*The pbe1* mutation repressed the expression of hypoxia-responsive genes. (**A**) GO analysis of downregulated DEGs (≤0.5-fold down compared to WT) showed that GO terms associated with hypoxia and biotic stimulus responses were enriched. The marked GO terms were also enriched in downregulated DEGs in *rpn10*, *rpt12a* mutants, or MG132-treated wild-type seedlings (see Table S3 for details). (**B**) Expression change heat maps of hypoxia-responsive genes in the *pbe1* mutant compared to WT. Red and blue represent the relative expression of genes among different samples in the same row. Three biological replicates were conducted for each sample. (**C**) The expression of several key hypoxia-responsive genes was detected by RT-qPCR. The expression level of each gene in WT was set to one. Data are the mean ± SD, n = 4. Significance analysis of differences was performed by *t*-test (** p < 0.01). (**D**) The interaction network of Arabidopsis proteins encoded by downregulated DEGs. The connections reflect known protein-protein interactions collected from the STRING database for downregulated DEGs. Nodes represent genes (downregulated DEGs), and edges represent the interaction between two genes. The size of a node is directly proportional to the degree of the node. That is the more edges connected to the node, the greater the degree of the node, indicating the stronger importance of genes in the network. The important nodes are mainly associated with hypoxia and biotic stimulus-response and are highlighted with red or yellow colors in the network.

Proteasome dysfunction also leads to the downregulation of many genes (Figure 2A). However, we are still unable to understand these downregulated genes. Here, we performed GO analysis of the downregulated DEGs in the *pbe1* mutant compared with the WT plant and found that the hypoxia- and biotic response-related GO terms were enriched (Figure 3A–C). The interaction network analysis showed that DEGs responding to hypoxia and biotic stress were located at central nodes of the interaction network (Figure 3D). Proteasomes play an important role in plant immunity, and plants with proteasome subunit mutations were shown to be hypersensitive to biotic stress [12,28,29,30]; thus, it is not surprising that plant immune genes are downregulated during proteasome dysfunction. However, interestingly, many hypoxia-responsive genes were also downregulated in *pbe1* mutants, and hypoxia-related GO terms were enriched (Figure 3). The downregulation of hypoxia response genes in the *pbe1* mutant was also confirmed by the expression heat maps and RT-qPCR analysis (Figure 3B,C). Meanwhile, our reanalysis of the transcriptome data from [27] also revealed that most of these hypoxia-related GO terms in the *pbe1* mutant were also enriched in the downregulated DEGs in *rpn10* and *rpt12a* mutants or MG132 (a proteasome inhibitor)-treated wild-type seedlings (Figure 3A and Table S3). In brief, transcriptome analysis of the *pbe1* mutant and other plants with proteasome dysfunction showed that proteasome dysfunction leads to inhibition of the hypoxic response pathway.

### 2.4. Arabidopsis PBE1 Mutants Are Hypersensitive to Waterlogging Stress

**Figure 4:**
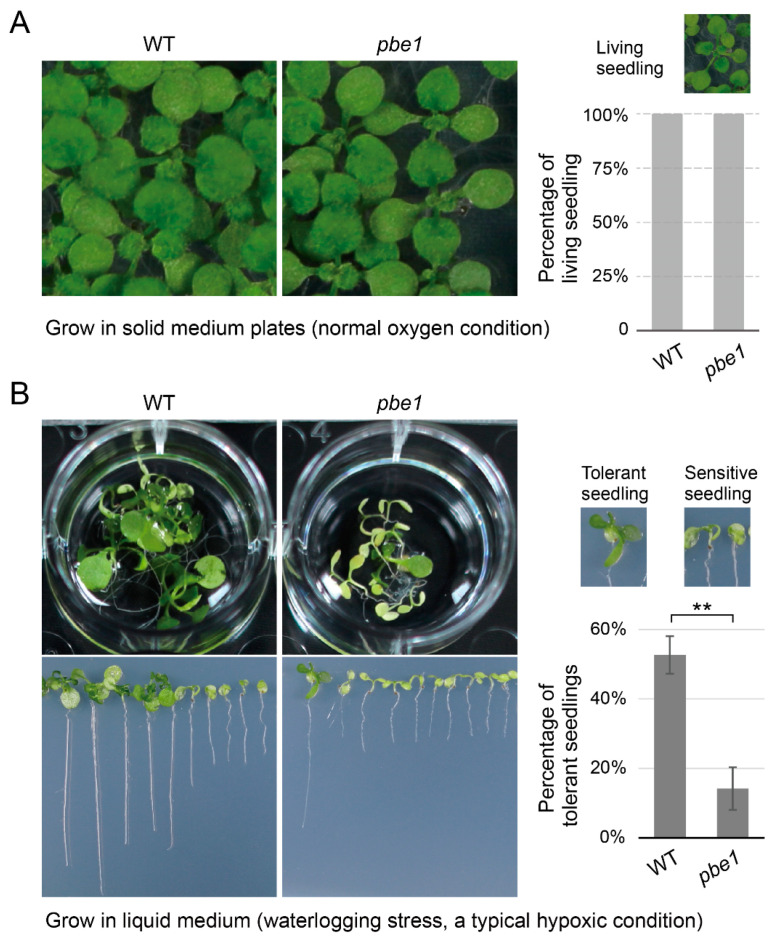
The *pbe1* mutant is hypersensitive to hypoxia stress. (**A**) WT and *pbe1* mutant plants grow in half-strength MS medium plates under normoxic (normal oxygen) conditions. Under normoxic conditions, the percentages of living seedlings of *pbe1* mutant and WT plants were both 100%. (**B**) The percentage of seedlings tolerant to waterlogging stress in WT plants was significantly higher than that in *pbe1* mutant plants. WT and *pbe1* mutant plants grow in plates for five days and then move to a liquid medium (waterlogging stress, a typical hypoxic condition) and grow for approximately 13 days. Under waterlogging stress, tolerant seedlings can produce true leaves, while sensitive seedlings have only cotyledons. Data are the mean ± SD. Significance analysis of differences was performed by *t*-test (** *p* < 0.01).

The hypoxia response pathway helps plants overcome hypoxic conditions, such as waterlogging stress [31]. We speculate that the downregulation of hypoxia response genes may impair plant tolerance to hypoxic conditions; thus, we first examined the tolerance of *pbe1* mutant plants to waterlogging stress (a typical hypoxic condition). As expected, although WT and *pbe1* mutant plants grew normally in plates at normoxic (normal oxygen) conditions, *pbe1* mutant displayed a highly sensitive phenotype to waterlogging stress compared to the WT (Figure 4). These results showed that *PBE1* mutation impaired the tolerance of plants to hypoxic stress, which is consistent with the transcriptome data that hypoxia response genes were downregulated in the *pbe1* mutant.

### 2.5. PBE1 Mutation Causes Hypoxia-Related Developmental Defects in Arabidopsis

#### 2.5.1. PBE1 Regulates Root Development

**Figure 5:**
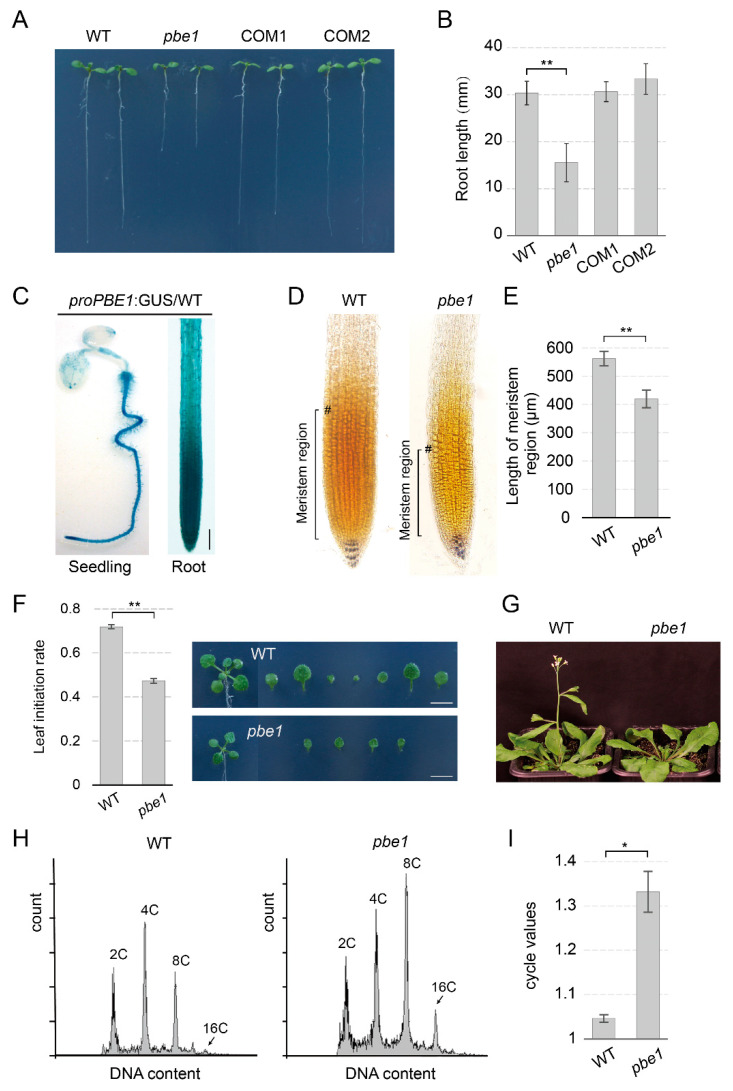
Development phenotypes of *pbe1* mutant plants. (**A**,**B**) Root elongation was inhibited in the *pbe1* mutant compared with the WT. Seedlings of WT, *pbe1,* and genetic complementation plants *proPBE1*:PBE1/pbe1 (COM1 and COM2) grow on one-half-strength MS medium plates. The root lengths of approximately 8-day-old seedlings were measured. Data are presented as the means ± SD (n ≥ 20). (**C**) GUS staining of *proPBE1*:GUS/WT seedlings shows that the expression of *PBE1* is predominately located in the root, especially the root meristem region. (**D**) Lugol’s staining of roots in WT and *pbe1* seedlings. The abnormal staining pattern of starch granules in the *pbe1* mutant suggests an abnormal root stem cell status in the *pbe1* mutant compared with the WT. (**E**) The meristem length of WT and *pbe1* seedlings in (**D**) was measured. Data are presented as the means ± SD (n ≥ 30). Significance analysis of differences was performed by *t*-test (** *p* < 0.01). (**F**) The *pbe1* mutant exhibited a decreased leaf initiation rate (leaves per day) as compared to the WT plant under normoxic (normal oxygen) conditions. Shoot phenotype and leaf number of 12-day-old WT and *pbe1* mutant plants that were grown in normoxic conditions. The leaf initiation rate was calculated by dividing the total number of leaves (the number of leaves on the twelfth day minus that on the sixth day) by the number of days (6 days). (**G**) The late flowering phenotype of *pbe1* mutants. The flowering of the WT and *pbe1* grown under long-day conditions. (**H**,**I**) The re-replication level is enhanced in *pbe1* mutants. The DNA ploidy content of cotyledons was analyzed by flow cytometry, and the cell cycle values of WT and *pbe1* mutant plants were calculated. The higher cycle values correspond to a greater re-replication level in the cotyledon of seedlings. Data are the mean ± SD. Significance analysis of differences was performed by *t*-test (* *p* < 0.05).

Recent studies have shown that hypoxia responses play an important role in plant development [31,32]. Overexpression of hypoxia-responsive genes can promote root elongation [33,34]; thus, we speculated that the downregulation of hypoxia-responsive genes might lead to suppression of root elongation in the *pbe1* mutant. As expected, root elongation under normal growth conditions was indeed inhibited in the *pbe1* mutant compared with WT plants (Figure 5A,B). The defective root development phenotype of the *pbe1* mutant was rescued in its genetically complementary plants (*proPBE1:PBE1*/*pbe1*) COM1 and COM2 (Figure 5A,B). The GUS staining of *proPBE1*:GUS/WT transgenic plants was mainly located in the root meristem region, suggesting that PBE1 may function in the root meristem (Figure 5C). It is well known that root elongation is derived from root meristem, and the status of root meristem can be detected by Lugol staining [35]. In the Lugol staining assay, *pbe1* roots showed irregular starch granules below the quiescent center (QC), indicating abnormal root meristem in *pbe1* mutant compared with WT plants (Figure 5D,E). These results show that the Arabidopsis proteasome PBE1 subunit plays an important role in normal root development.

#### 2.5.2. PBE1 Mutation Impaired the Activity of Shoot Apical Meristem

The activity of shoot apical meristem (SAM) is maintained by upregulating the hypoxia response pathway within the hypoxic niche in SAM [31,36]. Normal SAM activity is required for leaf initiation [36]. Recent studies have shown that inhibition of the hypoxic response in SAM impaired SAM activity and led to a decrease in leaf initiation rate (leaves per day) [36]. We found that compared with WT plants, the *pbe1* mutant had a lower leaf initiation rate (number of leaves per day) under normoxic (normal oxygen) conditions (Figure 5F). Meanwhile, the normal function of SAM is required for the subsequent induction of flowering [37]. We found that the *pbe1* mutant had a delayed flowering phenotype compared to that of WT plants (Figure 5G). These results suggest that *PBE1* mutation may impair SAM activity, which may be involved in the downregulation of the hypoxia response pathway in the *pbe1* mutant.

#### 2.5.3. PBE1 Mutation Enhances re-Replication

In Arabidopsis, re-replication is ubiquitous in leaf cells and is characterized by the continuous re-initiation of DNA replication resulting in increased DNA content without clearly recognizable genome doublings [38,39]. In human cells, adaptive responses to hypoxia lead to the arrest of DNA replication and the cell cycle and bypassing hypoxia-induced cell cycle arrest induces re-replication [40,41]. If the hypoxia response pathway in plants is also involved in re-replication, as in human cells, the downregulation of hypoxia response genes should facilitate re-replication in plants. As previously described, the re-replication level of Arabidopsis cotyledons can be calculated by the cell cycle value [38]. Here, we found that the cell cycle value of the *pbe1* mutant was remarkably higher than that of WT plants under normal growth conditions (Figure 5H,I), which suggests that PBE1 mutation enhances the re-replication level in cotyledons.

### 2.6. rpn1a Mutants Are Hypersensitive to Hypoxic Stress and Have Hypoxia-Related Developmental Defects

**Figure 6:**
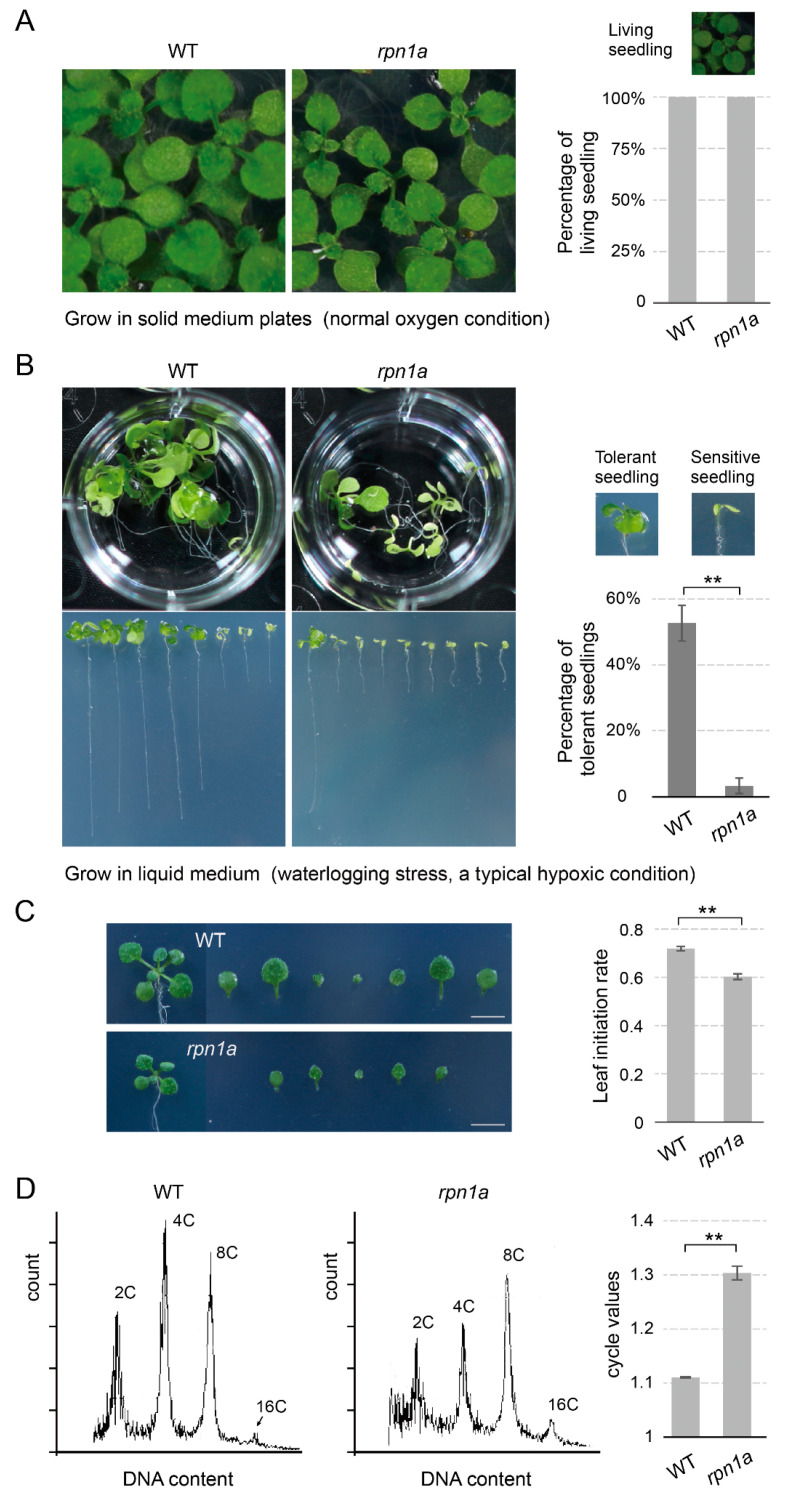
The *rpn1a* mutant is hypersensitive to hypoxia stress. (**A**) WT and *rpn1a* mutant plants grow in half-strength MS medium plates under normoxic conditions. The *rpn1a* mutant and WT plants had the same percentages of live seedlings. (**B**) The percentage of seedlings tolerant to waterlogging stress in WT was significantly higher than that in the *rpn1a* mutant. WT and *rpn1a* mutant plants grow on plates for 5 days and then move to a liquid medium (waterlogging stress, a typical hypoxic condition) and grow for approximately 13 days. Under waterlogging stress, tolerant seedlings can produce true leaves, while sensitive seedlings have only cotyledons. Data are the mean ± SD. Significance analysis of differences was performed by *t*-test (** *p* < 0.01) (**C**) The *rpn1a* mutant exhibited a decreased leaf initiation rate (leaves per day) as compared to the WT plant under normoxic (normal oxygen) conditions. Shoot phenotype and leaf number of 12-day-old WT and *rpn1a* mutant plants that were grown in normoxic conditions. The leaf initiation rate was calculated by dividing the total number of leaves (the number of leaves on the twelfth day minus that on the sixth day) by the number of days (6 days). (**D**) The re-replication level is enhanced in *rpn1a* mutants. The DNA ploidy content of cotyledons was analyzed by flow cytometry, and the cell cycle values of WT and *rpn1a* mutant plants were calculated. The higher cycle values correspond to a greater re-replication level in the cotyledon of seedlings. Data are the mean ± SD. Significance analysis of differences was performed by *t*-test (** *p* < 0.01).

RPN1a is an important 19S subunit and is required for embryogenesis and stress responses in Arabidopsis [12,13,14]. To explore whether the proteasome dysfunction caused by the 19S subunit mutation also impaired plant adaptability to hypoxia stress, we treated the *rpn1a* mutant with waterlogging stress (hypoxic condition). Similar to *pbe1* mutant, the *rpn1a* mutant displays a highly sensitive phenotype to waterlogging stress compared to the WT (Figure 6A,B). Meanwhile, the *rpn1a* mutant had a lower leaf initiation rate as compared to the WT plant (Figure 6C). In addition, the cell cycle value of the *rpn1a* mutant was also remarkably higher than that of WT plants (Figure 6D). Thus, these results showed that the proteasome dysfunction caused by 19S subunit mutations (such as *rpn1a*) also impaired the tolerance of plants to hypoxic stress and caused hypoxia-related developmental defects in Arabidopsis.

## 3. Discussion

The proteasome is a large complex composed of multiple subunits, and the loss of function of a single subunit (such as α7 subunit [PAG], β5 subunit [PBE], etc.) is often fatal to eukaryotes [2,4,24]. In Arabidopsis plants, PBE and many other subunits are mostly encoded by two paralogous genes [1], and their double mutants are usually lethal (e.g., PBE1/PBE2, RPT2a/RPT2b and RPN5a/RPN5b) [15,18,19,22]. This study further confirmed that the gametophyte and sporophyte of *pbe1 pbe2* double mutant are not viable, indicating that the PBE [β5] subunit is essential for the development of Arabidopsis. Although PBE1 and PBE2 are functionally redundant, the expression level of *PBE1* is much higher than that of *PBE2* [22]. Therefore, Arabidopsis *pbe1* single mutant caused proteasome dysfunction while keeping plants alive.

In plants, proteasome dysfunction has been shown to cause defects in plant growth and development and impair the plant’s ability to cope with environmental stress [26]. When the proteasome is inhibited or overloaded, the proteasome bounce-back response is activated to increase the expression of proteasome subunit genes [3,27]. In Arabidopsis plants, we showed that mutations in the 20SP subunit *PBE1* induced the proteasome bounce-back response and increased the expression of proteasome subunit genes, which is consistent with the results in 19SP subunit mutants and MG132-treated wild-type plants [27]. However, these results cannot explain the growth inhibition effect of proteasome dysfunction. In fact, in addition to the upregulated genes, a significant number of genes were downregulated in plants with proteasome dysfunction. Here, our transcriptome analysis showed that many hypoxia response genes are downregulated in the *pbe1* mutant, and hypoxia-related GO terms were enriched in the downregulated DEGs (*pbe1* divided by WT). Meanwhile, we reanalyzed the downregulated DEGs of the transcriptome data of 19SP subunit mutants or MG132-treated wild-type seedlings [27] and found that most of these hypoxia-related GO terms that were enriched in the *pbe1* mutant were also enriched in the downregulated genes of 19SP subunit mutants or MG132-treated wild-type seedlings. Furthermore, mutants of the 20SP subunit (such as *PBE1*) or 19SP subunit (such as *RPN1a*) both showed impaired adaptability to hypoxic stress. Thus, these results suggested that proteasome dysfunction leads to decreased expression of hypoxia response genes, which in turn impaired plant adaptability to hypoxia stress.

Oxygen sensing in plants is mediated by a branch of the N-end rule pathway that controls the stability of constitutively expressed ERF-VII transcription factors, which are primary activators of core hypoxia response genes such as *ADH, PCO, and SUS* [42,43,44]. The N-end rule pathway is a part of the ubiquitin-proteasome degradation pathway and consists of two branches, the Ac/N-end rule, and Arg/N-end rule pathways [45,46]. ERF-VII proteins accumulate under hypoxic conditions to activate the expression of hypoxic response genes but are continuously degraded by the proteasome through the Arg/N-end rule pathway under normoxic conditions [42]. If only based on this signaling pathway, proteasome dysfunction is expected to result in the accumulation of ERF-VII proteins, which in turn promote the expression of hypoxic response genes. However, this is not the case. Here, our data and our reanalysis of the data of previous studies showed that proteasome dysfunction leads to the suppression of hypoxia response genes (Figure 3). Therefore, we assume that, under normoxic conditions, there may be one or more hitherto unknown protein factors that specifically inhibit the expression of hypoxia-responsive genes and are degraded by the proteasome degradation pathway. When proteasome function is impaired or inhibited under normoxic conditions, these unknown proteins accumulate, which in turn suppress the expression of hypoxia-responsive genes. In future studies, the *pbe1* mutant and other plants with proteasome dysfunction will provide available plant material for the identification and functional analysis of this unknown protein.

In addition to hypoxia-responsive pathways, biotic stress response-related GO terms were also enriched in the downregulated (*pbe1* divided by WT) DEGs. The interaction network analysis showed that some important nodes belonged to both hypoxia and biotic stress pathways (Figure 3D), suggesting crosstalk between the two signaling pathways. Recent studies have found that the hypoxia signaling pathway plays an important role not only in plant development [31,32,36] but also in the interactions between plants and pathogenic microorganisms [47,48,49,50,51]. Meanwhile, mutants of proteasome subunits, such as *RPN1a*, are required for innate immunity in Arabidopsis [12]. Considering that the proteasome acts as a hub for plant immunity [30,52], we believe that the proteasome degradation pathway may play an important role in the crosstalk between plant immunity and adaptability to hypoxia stress.

Furthermore, the proteasome degradation pathway is crucial in all eukaryotic cells and organisms, and proteasome dysfunction is associated with diverse human diseases, including cancer [3]. In human cells, the proteasome is the target of many cancer therapies, and proteasome inhibitors are often used in anticancer therapy [2,3]. Recent studies have shown that the significant upregulation of hypoxic metabolism is a prominent feature of cancer cells [53], and hypoxia and 26S proteasome dysfunction cooperate to regulate immunity in human cells [54,55]. Thus, the inhibition of hypoxic response pathways caused by proteasome dysfunction may also be conserved in cancer cells.

## 4. Materials and Methods

### 4.1. Plant Material and Growth Conditions

*The* Arabidopsis *pbe1* mutant (SALK_092686, a loss-of-function mutant of *PBE1*), *pbe2* mutant (SALK_004669, a loss-of-function mutant of *PBE2*), *rpn1a* mutant (SALK_127430, a loss-of-function mutant of *RPN1a*), and methods for plant growth were described previously [12,22]. Arabidopsis wild-type (WT) and mutant seeds were sown on plates containing ½ MS solid medium with 1.5% sucrose, stratified at 4 °C for 3 days, and then grown under standard growth conditions (22 °C, 16 h light:8 h dark, 60% humidity) in growth chambers. For the treatment of waterlogging stress (a typical hypoxic condition), four-day-old seedlings grown on ½ MS solid medium were transferred to a cell culture plate containing ½ MS liquid medium without sucrose and then grown for 12 days.

### 4.2. Western Blotting

Two-, four-, six- and eight-day-old Arabidopsis seedlings growing on ½ MS solid medium were used for total protein extraction with Buffer F (50 mM Tris-HCl pH 7.5, 25 mM NaCl, 2 mM MgCl_2_, 1 mM EDTA, 2 mM dithiothreitol (DTT), 5 mM ATP, 5% glycerol). The extracted proteins were quantified by the Super Bradford Protein Assay Kit (CW0013S, CWBIO, Taizhou, Jiangsu, China) and then used for native polyacrylamide gel electrophoresis (PAGE) or sodium dodecyl sulfate-PAGE, followed by standard Western blotting. The anti-PAG1, anti-ubiquitin, and anti-actin antibodies were described previously [22].

### 4.3. Transcriptome and Reverse Transcription-Quantitative PCR Analysis

Six-day-old Arabidopsis seedlings growing on ½ MS solid medium were harvested for transcriptome and reverse quantitative transcription PCR (RT-qPCR) analysis. The methods for transcriptome and RT-qPCR were used and described previously [56]. Briefly, the extracted total RNA was used for sequencing with a NovaSeq 6000 sequencer by Majorbio (Shanghai, China). The clean reads were mapped to the A. thaliana TAIR10 database. Bioinformatics analyses of the transcriptome were performed using the online platform of Majorbio Cloud Platform (Shanghai, China). To identify DEGs between WT and *pbe1* mutant samples, the expression level of each transcript was calculated according to the transcripts per million reads (TPM) method. RSEM was used to quantify gene abundances. Differential expression analysis was performed using DESeq2 with Q-value ≤ 0.05. DEGs with an absolute log2 fold-change ≥ 1 and Q-value ≤ 0.05 were considered to be significantly differentially expressed genes (DEGs). Gene Ontology (GO) functional enrichment analysis of DEGs was carried out by Goatools. For RT-qPCR, the total RNA of WT or *pbe1* samples was extracted with TRNzol Universal Reagent (DP424, TIANGEN, Beijing, China). First-stand cDNA synthesis and qPCR were performed by using a One-Step gDNA Removal and cDNA Synthesis SuperMix kit (AT311, TransGen, Beijing, China) and SuperReal PreMix Plus (SYBR Green) kit (FP205, TIANGEN, Beijing, China), respectively. The expression of housekeeping genes *PP2A* (AT1G69960) or *ACTIN* (AT1G49240) was used as an internal control. All the primers are listed in Table S4.

### 4.4. Analysis of Plant Root Development

The genetic complementation plants *proPBE1*:PBE1/WT (COM1 and COM2) and the transgenic plants *proPBE1*:GUS/WT were described previously [22]. Arabidopsis seedlings were grown on ½ MS solid medium for approximately 8 days and analyzed for root phenotypes. Meanwhile, to measure the size of the meristematic root zone, seedlings of WT and pbe1 mutant plants were stained with Lugol’s solution (0.8 g potassium iodide was dissolved in 10 mL distilled water, then 0.1 g iodine was added to shake and mix and stored in the dark) and then photographed under the differential interference phase contrast microscope (DIC). GUS solution (1 mg/mL X-Gluc, 0.1 M sodium phosphate pH 7.0, 10% methanol) was used in the GUS staining analysis.

### 4.5. Flow Cytometry

WT and *pbe1* mutant Arabidopsis were grown on ½ MS solid medium for approximately 9 days and used for flow cytometric analysis according to [38]. Briefly, nuclear suspensions from cotyledons of WT or *pbe1* mutants were prepared using 1 mL extraction buffer (1×PBS, 10 mM MgCl_2_, 0.1% 2-mercaptoethanol, and 0.25% Triton X-100). The collected nuclei were suspended in 0.5 mL propidium iodide (PI) buffer (extraction buffer plus 1 µg mL^−1^ RNase A and 0.1 mg mL^−1^ PI), incubated at 37 °C for 30 min, and then directly used for flow cytometry analysis in a Sysmex Partec Ploidy Analyzer. The endopolyploidy cycle value was calculated using the following formula: Cycle value = [(n2C*0) + (n4C*1) + (n8C*2) + (n16C*3) + (n32C*4)]/(n2C + n4C + n8C + n16C + n32C), where n = the number of counts per a given C-value content.

## Figures and Tables

**Table 1:** *pbe1 pbe2* Double Mutants Are Inviable.

Genotypes of Progeny from Selfed AaBb Parent ^a^			
Genotype	Number ^b^	%	Expected % ^c^
AABB	12	15.8%	6.25%
AAbb	7	9.2%	6.25%
aaBB	8	10.5%	6.25%
AaBb	11	14.5%	25.0%
AABb	29	38.2%	12.5%
AaBB	9	11.8%	12.5%
aaBb	0	0	12.5%
Aabb	0	0	12.5%
aabb	0	0	6.25%

a AaBb is pbe1 pbe2 heterozygous double mutant. pbe1 is SALK_092686, pbe2 is SALK_004669. PBE1 (A), pbe1 (a), PBE2 (B), pbe2 (b). b Total individuals genotyped = 76. Genotypes of more progenies see Table S1. c Expected genotypes if all combinations are viable.

**Table 2:** *pbe1 pbe2* Double Mutants Are Gametophytic Lethal.

Genotypes of Progeny from AaBb ×WT Cross ^a^		
Genotype	AaBb Pollen × WT Egg Number	AaBb Egg × WT Pollen Number
AB × AB	57	87
aB × AB	33	58
Ab × AB	46	58
ab × AB	0	0
*Total*	*136*	*203*

a PBE1 (A), pbe1 (a), PBE2 (B), pbe2 (b). pbe1 is SALK_092686, pbe2 is SALK_004669.

## Data Availability

The data that support the findings of this study have been deposited into CNGB Sequence Archive (CNSA) [57] of China National GeneBank DataBase (CNGBdb) [58] with accession number CNP0003759.

## Supplementary Materials

The following supporting information can be downloaded at: https://www.mdpi.com/article/10.3390/ijms232416148/s1.
