# Supplementary material for: Proteasome Dysfunction Leads to Suppression of the Hypoxic Response Pathway in Arabidopsis
Source: Int J Mol Sci. 2022 Dec 18;23(24):16148. doi: 10.3390/ijms232416148 (PMC9785350; doi:10.3390/ijms232416148)
Supplement: Supplementary file 1 [file ijms-23-16148-s001.zip › Table S1 and S4 IJMS.pdf]

Supplementary Materials

Table S1. Pbe1 pbe2 double mutants are inviable

Genotypes of Progeny from Selfed AaBb Parent [a]			
Genotype	Number [b]	%	Expected % [c]
AxBy [d]	266	80.1%	56.25%
aaBB	35	10.5%	6.25%
AAbb	31	9.3%	6.25%
aaBb	0	0	12.5%
Aabb	0	0	12.5%
aabb	0	0	6.25%

[a] *PBE1* (A), *pbe1* (a), *PBE2* (B), *pbe2* (b). *pbe1* is SALK_092686, *pbe2* is SALK_004669.

[b] Total individuals genotyped = 332.

[c] Expected genotypes if all combinations are viable.

[d] x is A or a; y is B or b. AxBy includes AABB, AaBB, AABb, AaBb.

Table S4. All primers used in this study.

Gene	AGI ID	Name	5' to 3'
ACTIN8	AT1G49240	ACT8-qF	CCCGAGCAGCATGAAGATTA
		ACT8-qR	CTGAGGGAAGCAAGGATAGAAC
PP2A	AT1G69960	PP2A-qF	TATCGGATGACGATTCTTCGTGCAG
		PP2A-qR	GCTTGGTCGACTATCGGAATGAGAG
ADH1	AT1G77120	ADH1-qF	GAATCGCTGGTGCTTCTA
		ADH1-qR	CCTGTTGAATTGGCTTGTC
RGAT1	AT1G19530	RGAT1-qF	AAGAAGAATGAAGAAGAAGAACA
		RGAT1-qR	CTCCACCATCCACTACTC
CBP60G	AT5G26920	CBP60G-qF	AACACTTCTCTTCAACTCTG
		CBP60G-qR	CTGTAATGCGGTTAAGGTT
CYP81F2	AT5G57220	CYP81F2-qF	TCTTCATTGCCTCTCGTA
		CYP81F2-qR	ATAGTGGTGTAATCGTAAGC
ERF071	AT2G47520	ERF071-qF	GTAGAAGAAGAAGCCGATACTA
		ERF071-qR	TAATCCTCCAATGCCATCA
PCO1	AT5G15120	PCO1-qF	TGGTCCTGGTGTTATTCC
		PCO1-qR	GTTATTGGTGGCGAAGAC
PCO2	AT5G39890	PCO2-qF	CGGTTCTTGATGTTATTGGT
		PCO2-qR	CATAGCCTTCCTTCTCCTC
POX1	AT3G30775	POX1-qF	TTCTCGCAACACATAACG
		POX1-qR	GCATCTGACATACCATATAGC
SUS1	AT5G20830	SUS1-qF	GCAACAAGGACTCAACATTA
		SUS1-qR	GGCACACGAAGAATATCAC
SUS4	AT3G43190	SUS4-qF	GCATCTACTTCGCTTACAC
		SUS4-qR	TGGCTTCTTCTTGTCCTT
WRKY70	AT3G56400	WRKY70-qF	ACACCATCTCCGTTCTTG
		WRKY70-qR	TTGCCGTCGTTATCACAT
PAA2	AT2G05840	PAA2-qF	GTCCGAGGGAAAGATTCAGTATGC
		PAA2-qR	CCAGTGGCTAACAATCCAAGGT
PAG1	AT2G27020	PAG1-qF	TGGCTTGTCTCTGGCTTGA
		PAG1-qR	GTTCCAATGCTACTCATCTTCCTT
PBA1	AT4G31300	PBA1-qF	CTACTTCCTTCACCAGCATACAAT
		PBA1-qR	ATCCTCCAACGATGAGACCA
PBE1	AT1G13060	PBE1-qF	ATATGCTTACGGTGTGCTGGA
		PBE1-qR	CGGAATGTCGCATGGTAGATTG
PBE2	AT3G26340	PBE2-qF	GTTCAGGTTCACCATACGCTTAT
		PBE2-qR	CTTGCTAACTCTGAGGCTTCTTC
RPT2A	AT4G29040	RPT2A-qF	TGAGAGTTGTTGGTAGTGA
		RPT2A-qR	GAAGACGATTGATGGTGAA
RPN10	AT4G38630	RPN10-qF	CGCCTCACAGGAGACAGT
		RPN10-qR	TTCACATCACCAACAGACATAGC
RPN12A	AT1G64520	RPN12A-qF	TGAGTGGGAAGTGAAGGAAGG
		RPN12A-qR	GCTTAGAGTCTGGTTGATGAGTTG
